# Analysis of the fight against the COVID-19 pandemic in long-term care facilities in the pre-vaccination period

**DOI:** 10.1016/j.bjid.2024.103748

**Published:** 2024-05-04

**Authors:** Jáder Freitas Maciel Garcia de Carvalho, Rodrigo Ribeiro dos Santos, Alcimar Marcelo do Couto, Juliana Santos Neves, Flávia Lanna de Moraes, Cristiana Ceotto Deslandes, Viviane Rodrigues Jardim, Thiara Joanna Peçanha da Cruz Tavares, Raquel Souza Azevedo, Edgar Nunes de Moraes

**Affiliations:** a Universidade Federal de Minas Gerais, Faculdade de Medicina, Departamento de Clínica Médica, Belo Horizonte, MG, Brazil; b Universidade Federal de Minas Gerais, Hospital das Clínicas, Serviço de Geriatria, Belo Horizonte, MG, Brazil; c Prefeitura de Belo Horizonte, Secretaria Municipal de Saúde, Coordenação de Atenção Integral à Saúde do Adulto e Idoso, Belo Horizonte, MG, Brazil

**Keywords:** COVID-19, Long-term care facilities, Prevention, Pandemic, Health strategies

## Abstract

**Introduction:**

The COVID-19 pandemic has disproportionately affected individuals residing in Long-Term Care Facilities (LTCFs), necessitating tailored strategies to manage outbreaks. This study examines the outcomes of the ILPI BH project, a collaborative effort between the Municipal Health Department and the Hospital das Clínicas of the Federal University of Minas Gerais, designed to mitigate COVID-19 spread within LTCFs*.*

**Methods:**

Prospective cohort of secondary data: 1,794 old residents in 99 long-term care facilities of Belo Horizonte, Brazil, were followed from May 2020 to January 2021. The study analyzed the prevention strategies, residents’ clinical data, and the characteristics of the long-term care facilities, correlating these variables with the number of infections, hospitalizations, and deaths from COVID-19. It checked absolute numbers and rates of incidence, hospitalization, mortality, and lethality.

**Results:**

There have been 58 COVID-19 outbreaks in long-term care facilities. There were 399 cases among residents, 96 hospitalizations for COVID-19 and 48 deaths from COVID-19 (2.7 % of the cohort), with a case fatality rate of 12 %. After multivariate analysis, the intrinsic variables to residents associated with higher mortality risk were higher degree of frailty (OR=1.08; *p* = 0.004) and the fact of living in a long-term care facility with a considerable proportion of residents’ coverage by health plans (OR = 1.01; *p* = 0.028). Early geriatric follow-up showed an association with a reduction in the number of hospitalizations due to COVID-19.

**Conclusion:**

The correct classification of the degree of frailty of institutionalized older people seems to have been relevant for predicting mortality from COVID-19. The extensive assistance by private health plans, contrary to what is supposed, did not result in better health protection. Early geriatric follow-up was beneficial and may be an attractive strategy in the face of health emergencies that affect long-term care facilities to reduce hospital admissions.

## Introduction

The COVID-19 pandemic has precipitated significant challenges to individuals living in Long-Term Care Facilities (LTCFs). Following the declaration of the pandemic by the World Health Organization (WHO) in March 2020, reports emerged of severe outbreaks within LTCFs, particularly affecting the elderly.^1^, ^2^, ^3^, ^4^

Long-term care facilities are environments where frail older individuals predominate, with multiples morbidities. These factors increase the risk of developing severe forms of COVID-19.^5^, ^6^, ^7^, ^8^, ^9^, ^10^, ^11^, ^12^ Before the advent of vaccines, the lethality in older persons due to COVID-19 was around 14.8%,^12^ reaching about 50% for older people who needed hospitalization in Intensive Care Units (ICU).^5^^,^^13^ Some studies have assessed the average mortality rate from COVID-19 in the face of an outbreak in LTCF in about 25% to 33% of the total number of older residents infected.^7^^,^^8^

In Brazil, the National Health Surveillance Agency (ANVISA) prepared technical guidelines for measures to reduce the risk of transmission of COVID-19 in health services. It instructed health services to develop clear protocols to reduce the transmissibility of COVID-19, management of hospital clothing, and waste from health services.^14^

Although the LTCFs are not health units, it was necessary to create specific guidelines for this environment to reduce contagious by COVID-19. Isolation of residents with a suspected or confirmed case for 14 days in individual rooms was recommended and if it was impossible, the resident was should be transferred to an external service for testing and stay until the test result. Positive cases should stay out for 14 days from the first day of symptoms.^14^, ^15^, ^16^, ^17^

The Municipal Health Department (MHD) had previously identified LTCFs with structural or logistic problems to face the pandemic and classified those as highly vulnerable LTCFs. In order to prevent a disaster in the most vulnerable LTCFs to COVID-19, MHD and the Hospital das Clínicas of the Federal University of Minas Gerais (HC-UFMG) created the project entitled ILPI BH project aimed at monitoring and assisting LTCFs. It proposed to quickly identify and block COVID-19 outbreaks in LTCFs, with the key measure being the immediate transfer of LTCFs residents with suspected COVID-19 to a new care environment ‒ Provisional Reception Unit (UAPI) ‒ a non-hospital unit prepared to receive these people.

The objective of the present study was to analyze the results of this pioneering initiative and to understand the factors associated with a higher risk of incidence, hospitalization, and mortality from COVID-19, regarding the characteristics of residents and LTCFs, as well as the implementation of the proposed strategies*.*

As respiratory viruses are the principal causes of pandemics, this study may contribute to the understanding of the behavior of respiratory virus outbreaks in a LTCF and possible strategies that can facilitate planning for other health emergency situations in the future.

## Methods

This is a prospective cohort study, quantitative in nature. The data was daily extracted from the care records of the geriatrics service of the HC-UFMG, since the team that conducted the study coordinated the care work. The residents of the LTCFs registered in the ILPI BH project were followed from May 2020 to January 2021. HC-UFMG consented to use the data upon signature of the Data Use Agreement (DUA) by all researchers.

To help the LTCFs, which voluntarily joined the program, the geriatrics and gerontology service of the HC UFMG formed five multiprofessional teams composed of two Geriatric physicians, one nurse, and three to four physicians in training in geriatrics. Each team was responsible for monitoring and providing remote assistance to a group of LTCFs, together with the primary care of the Unified Health System (SUS). The first author of this study was one of the geriatricians responsible for the conference of the data collection and registration process and for conducting weekly meetings with the members for operational evaluation of the program and analysis of epidemiological data.

The geriatrics teams remotely organized and evaluated the information of all residents. The technical person in charge of the LTCF was asked to fill out a spreadsheet containing the following data: name, gender, date of birth, taxpayer's number (CPF), coverage (or not) by private health plan, degree of functionality by the Visual Frailty Scale (VFS),^18^ Degree of Resolution of the Collegiate Board of Directors (RDC) n° 502, of May 27, 2021,^19^ Clinical-Functional Vulnerability Index (IVCF-20).^20^ The IVCF-20 and the Visual Frailty Scale are instruments proposed by the Brazilian Ministry of Health.^21^ In addition, the technical person in charge of the LTCFs had to inform the lists of chronic morbidities and medications in use. The characterization of the study population was made from this information.

The inclusion and exclusion criteria in the study coincide with those of the assistance program:

Inclusion criteria: All residents of LTCFs registered in the ILPI BH project.

Exclusion criteria: Residents of LTCFs who do not participate in the ILPI BH project; persons whose LTCF has not sent the registration sheet.

The research implemented a communication network among the geriatric's teams, LTCFs, and primary care, using videoconferencing tools, e-mail, and an instant messaging application (WhatsApp®).

A specific online chatbot was created based on the clinical presentation of COVID-19 and its severity criteria for daily filling by the LTCF staff. This tool addresses five questions regarding the health status of the resident: if the axillary temperature is above 37.8 °C, if the heart rate is above 100 beats per minute, if the systolic blood pressure is below 100 mm of mercury (mmHg), or the diastolic blood pressure is below 60 mmHg, if the respiratory rate is above 28 respiratory incursions per minute, if there were symptoms (cough, dyspnea, odynophagia, anosmia, ageusia, prostration, mental confusion, or rhinorrhea). In case of a positive answer to any of the questions, the chatbot automatically sent an e-mail to the assistance teams, who made telephone contact to evaluate the case in detail. Chatbot generated spreadsheet reports of all response records performed.

The COVID-19 confirmed case criteria and the COVID-19 outbreak definition were based on the guidelines of the American Centers for Disease Control and Prevention.^22^ Thus, in the face of any upper airway symptom (cough, runny nose, dyspnea, sore throat, fever, anosmia, or ageusia), or even nonspecific symptoms, it was recommended that contact be made with the geriatrics team that remotely evaluated the case in order to determine if it was, in fact, a suspected case. In cases where there was a need, face-to-face medical care was provided to residents.

Confirmed COVID-19 cases were defined as those with a positive reverse transcriptase reaction test ‒ nasal swab, followed by Real-Time Polymerase Chain Reaction (RT-PCR).

The definition of a COVID-19 outbreak was the presence of one or more cases of COVID-19 among residents. Whenever there was a case with suspicion or confirmation of the disease, the geriatrics team contacted the primary care to ensure everyone was aware and could follow-up. A case confirmation entailed an RT-PCR testing of all other residents and employees of the LTCF. A case of confirmed infection in one or more employees also triggered all monitoring measures, including RT-PCR testing of all residents and other employees, as well as taking ten days off from work.

Once a resident was suspected of having contracted COVID-19, their immediate transfer to the UAPI was indicated, even before confirmation. The RT-PCR test was performed at the UAPI and, in case of two negative RT-PCR tests, with an interval of at least 24 hours between them, the resident returned to the LTCF. In case of a positive result, the resident was kept isolated at the UAPI for 14 days. There was also indication of transfer to the UAPI in the case of an asymptomatic individual with a positive RT-PCR test.

The study used the data on the number of patients admitted to the UAPI and their outcomes extracted from the digital epidemiological bulletin issued by Belo Horizonte City Hall (PBH) on February 1, 2021 (Annex 1).

### Independent variables

Character of LTCF: Whether the outbreak occurred in philanthropic or private for-profit LTCF.

Use of chatbot: A binary categorical variable was created to assess whether the LTCF team was using the chatbot the week before the outbreak began.

Early monitoring: The analysis considered early when the time difference between the onset of symptoms of the suspected case and communication with the geriatrics team was less than 48 hours. The follow-up means that the LTCF team solved its doubts and that there were guidelines to follow the program protocols, which included: guidelines for blocking the outbreak, sending an email to the Municipal Department of Health and Primary Care communicating the suspicion of the outbreak, requesting the transfer of the resident to the UAPI, guidelines for the isolation of the resident until a transfer, guidelines for the care of patients and other residents, such as: avoiding micro-nebulization, monitoring vital data and oximetry, attention to signs of severity.

Early transfer: When the transfer of the first suspected case occurred within 48 hours.

### Characteristics of residents

Categorical: Sex, nature of the LTCF in which they live (philanthropic or private for profit), individual coverage by private health plan, hypertension, diabetes, polypharmacy (defined as use of 5 or more drugs every day), degree of fragility by RDC n° 502/2021.^19^

Numerical: Age, number of residents in the LTCF where they live, clinical and functional frailty calculated by VFS and IVCF-20, proportion of coverage of residents of the same LTCF by health plans.

### Dependent variables

Ratings in absolute numbers: Number of COVID-19 infections, hospitalizations, and deaths among residents by outbreak; number of infections in employees.

Percentage assessments by outbreak: Incidence rate, mortality rate, and lethality were calculated.

### Statistical analysis

Descriptive analysis: The numerical variables were described in terms of median, first and third quartiles as none of them presented a normal Gaussian distribution. Categorical variables were described in terms of frequency and percentage. The Shapiro Wilk test was used to verify the hypothesis of normal distribution of numerical variables.

Univariate analysis: To verify the hypothesis of association between two numerical variables, Spearman's linear correlation coefficient was adjusted. When comparing a numerical variable without normal distribution between two groups, the non-parametric Mann-Whitney test was applied. Multiple comparisons were performed to verify the hypothesis of a significant difference between possible pairs of groups using the Mann-Whitney test and T-Student test. To verify the hypothesis of association between two categorical variables, Pearson's Chi-Square test was applied. All these evaluations were carried out at a significance level of 0.20, where those variables that presented a p-value lower than 0.20 were taken to the multivariate adjustment stage.

Multivariate analysis: The linear regression model was applied to numerical outcomes and the logistic regression model to binary categorical outcomes. All significant variables in the univariate stage were taken for multivariate adjustment, and those with a p-value greater than 0.05 in this regression adjustment stage were excluded. Therefore, only analyzes whose p-value is less than 0.05 are included in the results.

In compliance with resolution 466/2012, the Research Ethics Committee of UFMG approved the research project, and it is registered on Plataforma Brasil through the process of CAAE number (Certificate of Presentation for Ethical Consideration) 40666720.0.0000.5149.

## Results

The study included 1794 residents of the 99 LTCFs registered in the ILPI BH project; 27 are philanthropic, and 72 are private for-profit organizations. It corresponds to approximately 50 % of the population living in LTCFs in the city.

**Fig. 1:**
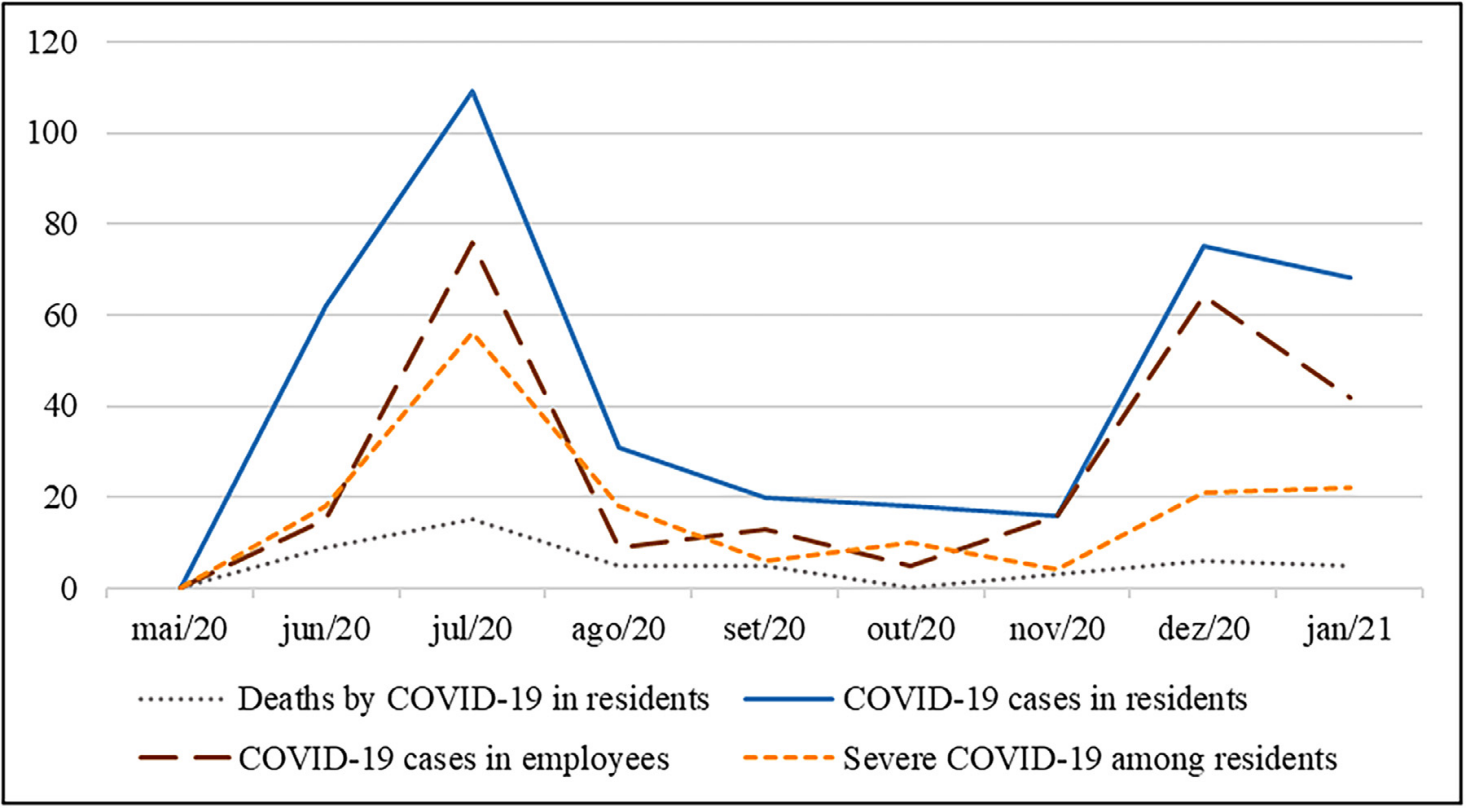
Epidemiological evolution of COVID-19 in the LTCF of the ILPI BH project. Note: The ordinate axis corresponds to the total number of epidemiological outcomes that occurred in each month.

At the beginning of the follow-up, no COVID-19 cases had yet been registered in any of the LTCFs followed. In the nine months of follow-up, there were 58 COVID-19 outbreaks, with 399 cases in residents, 96 of whom had to be hospitalized, and 48 died from COVID-19 (2.7 % of the cohort). The lethality rate of the virus in this cohort was 12.0 %. There were also 211 cases among employees. Fig. 1 shows the epidemiological evolution over the follow-up period.

There was only one LTCF that requested to leave the ILPI BH project after the first outbreak, which occurred in May 2020, because the LTCF coordination disagreed with the recommendations of the geriatric's teams. Thus, the study stopped accompanying the 13 residents in June 2020. It included the epidemiological data from this first outbreak; however, it is not known if there were other subsequent outbreaks in this LTCF.

Table 1 shows the general epidemiological data of COVID-19 outbreaks that occurred during the entire period studied.

**Table 1:** Epidemiological data regarding COVID-19 outbreaks in the cohort.

**COVID-19 outbreaks, n (monthly average)**	58 (6.4)
**Infections in residents, n (mean monthly)**	399 (44.3)
COVID-19 hospitalizations, n (monthly average)	96 (10.6)
Hosted at UAPI, n (monthly average)*	324 (36)
- Confirmed cases, n (monthly average)	158 (17.6)
- Confirmation ratio^a^ (%)	48.8
- Cases that have worsened^b^, n (monthly average)	60 (6.7)
- Deaths occurring in the UAPI	0
**COVID-19 deaths, n (monthly average)**	48 (5.3)
**Infections in employees, n (average monthly)**	211 (23.4)
**Case fatality rate (%)**	12.0

⁎ Source: Own authorship, except the data referring to the UAPI. Source: Epidemiological and care bulletins of the Municipal Health Secretariat of Belo Horizonte.

a Percentage of confirmed cases of COVID-19 among the total number of older people received in the period.

b Cases of COVID-19 or other pathologies that were isolated in the UAPI and had to be transferred to the UPA or hospitals due to worsening health condition.

Regarding the way in which the outbreaks were presented, 43 were discovered from a symptomatic older person; six from an investigation of a symptomatic employee; nine after universal screening testing (without symptoms).

In all outbreaks, after confirmation of the first case, there was testing with RT-PCR nasal swabs of all older individuals and employees (universal testing) for identification and isolation of asymptomatic and presymptomatic cases. In most of the outbreaks, universal testing was repeated ten days after the first test. However, there are no records that allow the research to identify in which of these 58 outbreaks this did not occur.

In analyzing the profile of LTCF residents, only 496 (27.6 %) of the total population had private health plans, with the rest being served exclusively by the public health system. While in philanthropic LTCFs, 10.5 % of residents have private health insurance, while in private LTCFs the percentage reaches 42.2 %. Table 2 also summarizes other data on the population profile in terms of clinical and demographic data.

**Table 2:** Characteristics of the study population and outcomes.

	Philanthropic	Private	Total	p
LTCF, n	27	72	99	
Residents by group, n (average per LTCF)	796 (29.4)	998 (13.9)	1794 (18.1)	
Median number of residents per LTCF (Q_1_‒Q_3_)	22 (17‒35)	14 (10‒16)	15 (11‒20)	<0.001^a^
Health Vulnerability Index, median (Q_1_‒Q_3_)	2 (1‒2)	1 (1‒2)	1(1‒2)	0.005^a^
Female sex	597	645	1242	<0.001^a^
Age, median	81	81	81	0.339^b^
VFS of the residents, median	8	8	8	0.663^b^
IVCF-20 of the residents, median	21	19	20	<0.001^b^
Classification by RCBD 502/2021, median	3	2	2	<0.001^b^
Prevalence of diabetes, n (%)	204 (25.6)	204 (20.4)	408	0.009^a^
Prevalence of systemic arterial hypertension, n (%)	498 (62.6)	534 (53.5)	1032 (57.5)	<0.001^a^
Coverage by health plans, n (average per LTCF in%)	69 (10.5)	427 (42.2)	496 (27.6)	<0.001^a^

Source: Own authorship.

VFS, Classification of fragility by the Visual Scale of Fragility; IVCF-20, Frailty Score by Clinical-Functional Frailty Index; Q_x_, N° of quartile.

Note: The p-value refers to the probability of significance of the Mann-Whitney (^b^) or Chi-Square (^a^) test.

Table 3 shows the number and proportion of outbreaks in which each of the strategies could be implemented, both in philanthropic and private for-profit LTCFs.

**Table 3:** Proportion of success in implementing the strategies of the ILPI BH project protocol and outcomes.

	Philanthropic	Private	Total
Outbreaks, n (%)	33 (56.9)	25 (43.1)	58 (100)
Early Geriatric Care, n (%)	15 (45.4)	4 (16.0)	19 (32.8)
Early transfer to UAPI, n (%)	6 (18.2)	4 (16.0)	10 (17.2)
Transfer to UAPI, even after 48 h, n (%)	24 (72.7)	12 (48.0)	36 (62.1)
Use of chatbot on the eve of the outbreak, n (%)	14 (42.4)	10 (40.0)	24 (41.4)
Incidence of COVID-19 among residents, n (%)	253 (31.8)	146 (14.6)	399 (22.2)
Hospitalizations due to COVID-19, n (average%)	50 (6.2)	46 (4.6)	96 (5.4)
COVID-19 deaths, n (mean%)	28 (3.5)	20 (2)	48 (2.7)
Incidence of COVID-19 among employees, n	170	41	211

Source: Own Authorship.

Table 4 shows the association between death from COVID-19 and some of the characteristics of the older individual infected by COVID-19. The result indicates that with each 1-point increase in the IVCF-20, the risk of death increased by 7.6 %. In addition, the higher the percentage of coverage of private health plans among older residents in the same LTCF as the infected older person, the higher the risk of death of this older person, and for every 1 % more coverage, the risk of death from COVID-19 increased by 1.2 %.

**Table 4:** Characteristics of infected residents and their association with the risk of death from COVID-19.

Characteristic of the resident	Univariate Analysis		Multivariate Analysis	
	Death from COVID OR (95 % CI)	P	Death from COVID OR (95 % CI)	p
Lives in a philanthropic LTCF	1.04 (0.54; 2.0)	0.91	‒	‒
Female sex	1.4 (0.71; 2.75)	0.33	‒	‒
Health Plan	1.68 (0.79; 3.55)	0.171	‒	‒
Diabetes	1.18 (0.58; 2.42)	0.647	‒	‒
Systemic Arterial Hypertension	1.02 (0.53; 1.96)	0.954	‒	‒
Polypharmacy	1.33 (0.66; 2.7)	0.428	‒	‒
Number of residents in the same LTCF	1.00 (0.99; 1.02)	0.653	‒	‒
% Coverage by health plans^a^	1.01 (1.01; 1.02)	0.034	1.012 (1.001; 1.024)	0.028
Age	1.03 (0.99; 1.06)	0.088	‒	‒
Fragility by VFS^b^	1.28 (1.01; 1.61)	0.041	‒	‒
Fragility by IVCF-20 score^c^	1.07 (1.02; 1.13)	0.005	1.076 (1.023; 1.132)	0.004
Fragility for the DRC 502/2021	1.06 (0.56; 2.03)	0.851	‒	‒
Chatbot usage ratio by LTCF	0.99 (0.99; 1.00)	0.179	‒	‒

Source: Own Authorship.

a Percentage of elderly covered by health plans, among the total number of older people living in the same LTCF as the infected elderly.

b Visual Frailty Scale.

c Clinical Functional Vulnerability Index. Note: Sample unit: individuals living in LTCF who were infected by COVID-19 (*n* = 343).

Although there have been 399 cases of COVID-19 among residents, the number of infected is 388 (since there have been 11 cases of re-infection). The database allowed us to identify only 343 of these residents. In the remaining 45 cases, although there is the registration of the outbreak date, the LTCF, and the number of positive RT-PCR results among the older people and employees, there is no name (or it is incomplete) that allows the identification of which older individual was infected.

Table 5 presents the analysis of the outcomes evaluating the association between the characteristics of the LTCF (character and number of residents) and strategies implemented, with the epidemiological outcomes.

**Table 5:** Univariate analysis of variables related to LTCFs and the strategies used.

Variables	Outcomes Correlation coefficient (95 % CI)			
	N° of Infections (residents)	Number of admissions (residents)	Number of Deaths (residents)	N° of infections (employees)
Philanthropic LTCF	0.16 (−0.12; 0.46)	−0.12 (−0.41; 0.19)	0.12 (−0.18; 0.4)	0.4^a^ (0.11; 0.65)
N° of residents	0.34^a^ (0.06; 0.58)	−0.19 (−0.47; 0.1)	0.19 (−0.13; 0.5)	0.41^a^ (0.11; 0.67)
Use of chatbot before the outbreak	−0.07 (−0.36; 0.22)	−0.13 (−0.44; 0.21)	−0.16 (−0.44; 0.12)	−0.18 (−0.44; 0.13)
Early Transfer	−0.18 (−0.5; 0.2)	−0.43^a^ (−0.62; −0.21)	−0.11 (−0.34; 0.17)	−0.02 (−0.34; 0.32)
Early geriatric follow-up	0.07 (−0.27; 0.32)	−0.44^a^ (−0.64; −0.19)	−0.19 (−0.42; 0.09)	0.19 (−0.15; 0.51)

Source: Own Authorship.

a p-value < 0.05. Note: Univariate analysis; sample unit: COVID-19 outbreaks; for the analysis of the categorical variable referring to the character of the LTCF, the value “zero” was assigned for private LTCF and “one” for philanthropic LTCF.

Table 6 presents the results of the multivariate analysis by linear regression obtained through the results shown in Table 5. For every ten more residents, per LTCF, about one more employee was infected per outbreak. In addition, early geriatric follow-up was associated with a reduction of about two hospital admissions per outbreak.

**Table 6:** Multivariate analysis of variables related to LTCFs and the strategies used.

Associated outcome	Variable	Beta (95 % CI)	Standard error	p
Number of infections among employees	N° of residents per LTCF	0.11 (0.06; 0.17)	0.28	<0.001
Number of hospitalizations among residents	Early geriatric follow-up	−1.98 (−3.78; −0.17)	0.89	0.032

Source: Own Authorship.

Notes: Linear regression analysis; sample unit: COVID-19 outbreaks; period: May 2020 to January 2021.

## Discussion

This is an unprecedented study in Brazil due to the cohort size, logistics employed, and associations found. Mortality from COVID-19 in the cohort was lower than that observed in the first months of the pandemic among institutionalized older adults in many countries. In October 2020, (about 9 to 10 months of the pandemic in Europe and the United States) the mortality rate from COVID-19 in the LTCF environment in some developed countries, such as Belgium, Spain, Sweden, England, Scotland, and the United States, had been, respectively, 5 %, 6.2 %, 3.3 %, 5.2 %, 5.6 %, and 4.2 %^1^^,^^24^. In the cohort of the ILPI BH project, analyzing the first ten months of the pandemic, mortality was 2.7 %.

According to the PBH epidemiological bulletin of February 1, 2021 (end of the period of the present study), there had been 2264 deaths from COVID-19 in Belo Horizonte, with only 2.1 % occurring in the studied population.

There are no other cohort studies that have included this number of residents of LTCFs and characterized their frailty profile and health conditions in Brazil. The combined monitoring strategies, remote and face-to-face, proved effective for the follow-up of these 1794 residents. The association between early geriatric follow-up and the reduction in the number of hospitalizations reinforces the importance of the measure since hospital beds were scarce during many of the pandemic periods. A decisive factor for success was the uniformity of conduct in the face of outbreaks, achieved from a protocol followed by all geriatric teams ‒ trained and monitored throughout the period by the program coordination.

At the beginning of the follow-up, there was an estimate of a higher mortality rate in the cohort, not only due to the high prevalence of frailty and the vulnerable profile of the institutions, but also due to the severity of the outbreaks reported in LTCFs of many of the countries. Although the design of the study does not allow us to affirm the reasons that led to this relative low mortality – when compared to that observed in developed countries - some hypotheses can be raised, and it is fair that this be done since it will not be possible to carry out clinical trials that elucidate these causes since it is not possible to repeat the same pandemic condition.

The climatic factor is a point to consider. Most scientific papers that have discussed COVID-19 in LTCFs in the initial months of the pandemic come from countries in the northern hemisphere and, therefore, countries that dealt with the virus in winter.^25^ The form of transmission of the virus, mainly by droplets but also by means of aerosols indoors, favors its spread during cold climates since they promote environments with reduced air circulation, especially in countries with temperate climates.^25^^,^^26^

The fact that the virus reached Belo Horizonte only in March 2020, three months after the start of the epidemic in Wuhan (China), allowed studies, guidelines, and protocols to be conducted, even before the first case was confirmed.^14^^,^^27^ The very advent of RT-PCR testing and the discovery of the effectiveness of the systematic use of masks favored the countries subsequently affected by the virus since they could count on these resources to prevent outbreaks that could have occurred without these measures. Non-pharmacological infection prevention measures are recognized to be valuable in reducing the risk of COVID-19 infection.^23^^,^^28^, ^29^, ^30^

Another relevant hypothesis is that the preliminary results of the RECOVERY study were published in June 2020, demonstrating a reduction in the lethality of the virus in patients with severity criteria who used dexamethasone.^31^ Until then, the disease did not have any effective pharmacological treatment, and the fact that dexamethasone is a quite common, cheap, and available drug, it soon began to be widely prescribed for the for severe COVID-19.

The possibility of universal testing with RT-PCR in all outbreaks is another fact that deserves attention. In addition to adding reliability to the epidemiological data, it allowed the diagnosis, with the gold standard examination, even of asymptomatic and oligosymptomatic older individuals. Studies have demonstrated the importance of universal testing to reduce the incidence and mortality of the disease.^28^^,^^30^

A data that requires further investigation was the fact that living in LTCFs with a high proportion of coverage by health plans was associated with higher mortality among the infected older people. It is necessary to consider the possibility that the private LTCFs, because they have a significantly higher percentage of residents covered by private health plans, have resorted more to private assistance than the activation of geriatrics teams of the ILPI BH project. In fact, as demonstrated, philanthropic LTCFs had greater adherence to the proposed preventive strategies.

Regarding the analyses carried out to identify the factors associated with a higher risk of death among the infected older individuals, it is interesting to note that factors linked to greater lethality of the virus in adults, such as advanced age, hypertension, and diabetes,^12^^,^^32^^,^^33^ were not relevant in this cohort, while the degree of frailty, when measured by IVCF-20, demonstrated association. Although the association between frailty and risk of death has already been widely demonstrated,^34^, ^35^, ^36^ even in the case of COVID-19,^6^ the association between the score obtained by the IVCF-20 and the risk of death from COVID-19 is unprecedented. The instrument was superior to the functional assessment by the VFS to predict the risk of death, while this was superior to the classification required by RDC n° 502/2021,^19^ which is the classification that regulates the functioning of the LTCFs in Brazil.

Although chatbots have been used as an aid to health services in the pandemic in some parts of the world and, in some experiences with satisfactory results,^37^^,^^38^ there was no association between its use and epidemiological outcomes in the present study.

The group of philanthropic LTCFs, even having a smaller population, had almost four times more cases of COVID-19 among employees. Although the study design does not allow causal inferences to be made, it is possible that the number may be underreported in the group of private LTCFs. The study found that some private LTCFs were reluctant to report outbreaks early and test mild symptomatic cases because of the possible economic impact since an employee with COVID-19 needed to be away from work for two weeks. In addition, it also noted that the discourse of some LTCFs leaders demonstrated the fear that the detection of a COVID-19 outbreak could generate negative publicity for their LTCF in the community. The association found between the number of infections among employees and the number of residents of the LTCF may be related to this fact since the median number of residents of philanthropic LTCFs is considerably higher than that of private for-profit LTCFs.

The main limitation is the absence of external comparability because there are no published data on COVID-19 outcomes in the other LTCFs in Belo Horizonte, whose management of outbreaks did not follow the protocol of the ILPI BH project. There may be inherent benefits to the entire cohort because there is a follow-up, frequent instructions, and support, which were not revealed by this absence of external comparison. There are also no other similar studies conducted in other cities in Brazil at the same time.

The difficulty in knowing the total number of LTCFs and institutionalized people, in Belo Horizonte and in Brazil, denotes a sociopolitical problem that deserves attention. This fact brings limitations since it is not possible to guarantee the proportion that the sample represents over the total population. To write the article, the author contacted the transparency portal of the city of Belo Horizonte, requesting information. However, they only informed about the number of philanthropic institutions partnered with the municipality, which is 24. The estimate that the cohort included around 50% of all LTCFs residents in the municipality was based on meetings with representatives from the municipal health and social assistance departments, who also recognized the difficulty in obtaining accurate and up-to-date figures.

Another limitation is that the sample characterization was done by filling out spreadsheets sent by e-mail, which interferes with the quality of the information because there was no direct evaluation of the cohort members for the data recording. It would also be essential if the study had assessed other factors, such as schooling, income, ethnicity, and prevalence of other risk factors, such as obesity and pneumopathies, but these data were not available.

## Conclusion

The monitoring and assistance provided by the geriatrics teams was a significant measure to support the LTCFs and to reduce hospital admissions in the pandemic. The correct measurement of frailty, made by the IVCF-20, can help predict the risk of evolution to death by COVID-19. Having good coverage of residents by private health plans did not result in better protection for LTCFs residents. On the contrary, the support provided by SUS through the ILPI BH project was associated with more satisfactory outcomes. The experience of the ILPI BH project can be useful for planning public policies to face future health emergencies, including pandemics.

## Conflicts of interest

The authors declare no conflicts of interest.

## Appendix Supplementary materials

Image, application 1
