# Supplementary material for: Analysis of the fight against the COVID-19 pandemic in long-term care facilities in the pre-vaccination period
Source: Braz J Infect Dis. 2024 May 4;28(3):103748. doi: 10.1016/j.bjid.2024.103748 (PMC11101719; doi:10.1016/j.bjid.2024.103748)
Supplement: Supplementary file 1 [file mmc1.doc]

BJID-D-23-00318_Supplementary Material

**Annex 1** Epidemiological bulletin of February 1, 2021.

| **TEMPORARY RECEPTION SERVICE FOR THE OLD PEOPLE – UAPI** | | |
| --- | --- | --- |
| CHART 8 – Temporary care service for the old people | | |
| **Old people** | **Nº** | **%** |
| Welcome until 01/29 | 324 | 100 |
| In reception at the moment | 33 | 10.19 |
| Transferred to other health services | 60 | 18.52 |
| Return to LTCF | 231 | 71.30 |
| Total positives | 158 | 48.77 |
| Observation: Unit for the isolation and adequate care of the elderly with mild respiratory symptoms without indication of hospitalization of the partner LTCFs of Belo Horizonte. SMSA/SMASAC partnership. | | |
| Source: PBH/SMSA-BH-updated 02/01/ 2021. | | |
| Source: Belo Horizonte City Hall, link: https://prefeitura.pbh.gov.br/saude/coronavirus. | | |
